# Making family medicine work: Rural community-based and interprofessional medical education

**DOI:** 10.4102/phcfm.v16i1.4583

**Published:** 2024-07-29

**Authors:** Dirk T. Hagemeister

**Affiliations:** 1 Department of Family Medicine, Faculty of Health Sciences, University of the Free State, Bloemfontein, South Africa; 2 Department of Family Medicine, Free State Department of Health, Bloemfontein, South Africa

**Keywords:** community-based education, family medicine, interprofessional education, rural, nurse-driven primary care, undergraduate medical education

## Abstract

At the University of the Free State, the 5-year MBChB curriculum had to be complemented with community-based education exposure to meet the requirements of the Health Professions Council of South Africa. Following the faculty leadership’s vision, an interprofessional training experience was conceptualised and implemented by a project team from the three schools in the Faculty of Health Sciences (Medicine, Nursing, and Health and Rehabilitation Sciences). For the past decade, 4th-year medical students participated in the 2-week rotation in the rural southern Free State province, of which 1 week is spent with students from other health professions programmes in a structured interprofessional learning experience. The other week focuses on the realities of nurse-driven primary healthcare services in a resource-deprived area, including exposure to the programme-guided care for patients with tuberculosis (TB) or chronic diseases, care for pregnant women and for babies, including vaccinations.

## Introduction

At the University of the Free State (UFS), undergraduate medical education has pursued innovative routes since the establishment of the Faculty of Medicine in 1978.^1^ In their classical paper, White et al. demonstrated that the overwhelming majority of patient encounters occur in an ambulatory context, mostly at primary healthcare (PHC) level.^2^ Traditionally, medical education takes place at academic medical centres, providing the full range of medical specialities and subspecialities.^3,4^ Medical students thus often learn their trade ‘the wrong way around’, starting their clinical journeys by clerking patients with rare diseases who have been exposed to maximum diagnostic resources, rather than by managing patients with undifferentiated complaints. To equip future practitioners with the skills required in their future generalist, PHC practice of medicine, the Health Professions Council of South Africa (HPCSA) requires undergraduate medical programmes to include community-based education.^5^

## Setting and intervention

Around 150 medical students graduate annually from the UFS’s 5-year undergraduate programme, with the numbers gradually increasing in keeping with the national trend. Universitas Academic Hospital, located adjacent to the main campus of the university in Bloemfontein, and Pelonomi Tertiary Hospital in the Mangaung township, are traditionally the main clinical training platforms, together with National District Hospital and community health centres in Bloemfontein.^6^

More than a decade ago, the university, guided by the vision of the dean of health sciences, acquired the premises of an out-of-business hotel in Trompsburg in the Xhariep District. As one of the five district municipalities in the Free State Province, the Xhariep District in the south of the province between the Mangaung Metro District of Bloemfontein and the Xhariep (Oranje) River is the largest and at the same time the least-densely populated district in the province, with a population of 135 000.^7^

## Our plan: Primary healthcare, community-based and interprofessional education

In 2012, a small project group for community-based education came together. Interprofessional from the beginning, it comprised one lecturer each from nursing, occupational therapy and family medicine. Engaging with a fallow training ground, there were no clear directions to follow. Thus, a wealth of community engagement and pilots followed. All along, the shared understanding between the lecturers involved was that the future training needed to expose the students to the realities in impoverished rural communities to complement hospital-centric training provided in most parts of the undergraduate programmes. As the new Albert Nzula District Hospital in Trompsburg was slow to become operational, the obvious choice for the medical students was placement in the PHC clinics.

In addition to the community-based PHC approach, the opportunities offered by the interprofessional planning team were to translate into interprofessional exposure at the grassroots level.

## The way we got there

When the medical programme eventually started using the expanded platform including the southern Free State, a 2-week rotation in Xhariep was the first step. Fourth-year medical students would spend 1 week in an interdisciplinary team with students from the other schools (Nursing and Health and Rehabilitation Sciences, typically 8–12 students from two to four disciplines) and a 2nd week with the PHC team in one of the two local clinics in Trompsburg and Springfontein.

Initial steps were undertaken while the hotel-turned-residence was still awaiting completion, using bed-and-breakfast accommodation. On completion, 72 beds became available in the residence to accommodate students. This constituted a massive investment on the side of the university. Continued funding for maintenance and security, employment of drivers for the university-owned vehicles, gardening and cleaning staff provides economic opportunities in this economically deprived area. This gain is complemented by the day-to-day spending of the visiting students and their tutors even though the full capacity for student placements still is to be utilised.

Considering the content of the 2 weeks, two separate but related programmes were designed. Driven by the interprofessional team, the interprofessional week would focus on activities in the community, reaching into schools and people’s homes. Activities include screening at the local high schools and comprehensive follow-up projects for the elderly and patients with chronic conditions in the community. The school health project incorporates and expands on the tools used by the government’s school health programme to assess the physical and mental well-being of the learners.

A support programme for patients with chronic diseases was initiated to follow up community members with diabetes or hypertension at their homes. This programme, again, offers the interprofessional student group another opportunity to apply their complementary skills to the benefit of community members.

Led by the occupational therapy team, both the school health and chronic care programmes involved dedicated support groups developed for the respective target populations. Learners from the three local high schools in the area were invited to join a youth leadership programme, and the chronic care clinic attendees at the two primary care clinics were invited to join health clubs offering health education and social contact. Both support groups are run by the students rotating on the platform, supported by the locally appointed university lecturer.

To round off the interprofessional experience, group tasks were added. These include a preparatory session on the main campus preceding the placement for the students to get to know each other while discussing a simulated patient with rehabilitation needs. Once booked into the residence, the students have a further discussion about the roles of the different professions and the mutual perceptions. By the end of the week, the interprofessional groups submit a digital story, comprising a photographic diary of their journey through the week, facilitating self-reflection.^8^

For the 2nd week, the Department of Family Medicine chose an entirely ambulatory care-based approach. The students are divided between the two PHC clinics and participate in the general PHC provided at these clinics. A comprehensive logbook guides this exposure, introducing nurse-driven services, including healthy baby vaccination clinics, care for patients with both acute and chronic conditions, basic antenatal care and the infectious diseases programmes for patients with tuberculosis and HIV. On different days during the week, the students are assigned to different areas in the clinic, including outreach to the local township with a community health worker or on the UFS-sponsored mobile clinic. The focus for this week lies on comprehensive, nurse-driven PHC services, deliberately leaving out doctor-provided activities in the hospital. To sustain this nurse-tutored learning opportunity, buy-in from the PHC staff was essential, a challenge mitigated by the involvement of the School of Nursing in the bigger project.

## Challenges and successes

A first challenge was the geographic location of the project with the associated logistical needs. Although the accommodation had been created, transporting approximately 8–20 students (depending on the undergraduate programmes involved in a given week) to Trompsburg, and from there to PHC clinics, schools and community members’ homes require complex arrangements and result in significant costs.

Supervision and training are provided by a combination of staff appointed for the platform (an occupational therapist as a university lecturer and a residence head with supporting staff) and additional visiting lecturers from the three schools of the Faculty of Health Sciences. Each of the latter spends 1 week on the platform assisting the former when students are split between the towns of Trompsburg and Springfontein.

Given the busy schedule at academic institutions, the availability of staff (and students) constitutes an ongoing challenge. Frequently, several requests are needed to populate the slots for visiting lecturers through the year.

Possibly even more demanding is the coordination of placement of the undergraduate students on the platform to allow for interprofessional experience. With the medical (MBChB) programme being the largest in the faculty, medical students vastly outnumber the students in other programmes such as Nursing, Occupational Therapy, Physiotherapy, Optometry and Nutrition and Dietetics. The School of Clinical Medicine is placing students on the platform in 18 2-week blocks over the year, thus being present for 36 weeks of the year. The other undergraduate programmes, smaller in student numbers, have a more limited presence, with the School of Nursing initially having had 18 weeks and some other programmes only joining 1 or 2 weeks per year.

This resulted in a random and unpredictable mix of undergraduate students and skills in any given week. When no other programme’s students are available to work with the medical students on the platform, the so-called ‘quasi-IPE’ weeks try to replicate the interprofessional education (IPE) experience with the medical students also covering the tasks tailored for other disciplines. At times, it constitutes a rewarding challenge for other professions to fill the gaps while using tools initially included to accommodate a specific programme such as Optometry, Dietetics or Physiotherapy.

It would be dishonest to ignore the ‘attitude issues’ of both the students and the academic staff in the faculty. Academic clinicians tend to regard time spent on a rural and PHC-focused rotation as a waste of academic time that could, given the packed 5-year curriculum, be better spent on ‘real’ clinical practice. Although such opinions are rarely openly expressed, they fuel the unwillingness to expand the ‘southern Free State experience’ into other phases of the MBChB programme. Similarly, and most likely influenced by this attitude, is the occasionally expressed notion of the undergraduate medical students that they ‘cannot really learn anything in the southern Free State’.

On the contrary, many highly appreciative comments complimenting both the rural PHC and especially the interprofessional experience rewarded the lecturers involved plentifully. Typical terms used by the students in their reflections included ‘skeptical’, ‘intimidating experience’ and ‘unfamiliar experience’ but also ‘opened our eyes’ and ‘integrated deep wealth of knowledge’, as published by our group before.^8^

## Conclusion

### Reflections and summary

In brief, it was, and still is, an amazing journey to create this unique learning opportunity for our students in an otherwise very science-focused and biomedical curriculum. Exposure to rural PHC with a strong interprofessional perspective was established. This led in the following years to an expansion of the initial 2 weeks to a 4-week module, adding a further 2 weeks at the district hospital and PHC clinics in the Botshabelo area east of Bloemfontein.

### What remains to be done?

Given the 5-year duration of the academic programme, the current exposure of merely 4 weeks during the 4th year, plus an additional 2 weeks at a community health centre in the 5th year, remains very little. Attempts to establish an earlier exposure in a pre-clinical function, for example, to provide community-mapping and household surveys by early-phase medical students, have been unsuccessful so far. Of concern regarding the sustainability of the programme is support for the local PHC staff in the southern Free State, who with great commitment are providing a unique learning experience for year after year of medical students. This should deserve, besides explicit recognition, a significant reward from the UFS, which could, for example, be in the form of free access to academic modules in nursing education.
